# Inference in group sequential designs with causal mechanisms: implications for power and mediation analysis

**DOI:** 10.1186/s12874-025-02714-y

**Published:** 2025-11-14

**Authors:** Kim May Lee, Richard Emsley

**Affiliations:** Department of Biostatistics and Health Informatics, Institute of Psychiatry, Psychology and Neuroscience, King’s College London, 16 De Crespigny Park, SE5 8AF London, UK

**Keywords:** Mediation analysis, Group sequential designs, Direct effect, Indirect effects, Conditional maximum likelihood estimator, Penalized maximum likelihood estimator

## Abstract

**Background:**

Group sequential designs are increasingly employed to allow trials to stop early with statistical rigor. While existing work focuses on intention-to-treat effect on clinical endpoints, the properties of mediation analysis (commonly conducted in psychological trials to understand a causal mechanism) remain unknown under group sequential designs.

**Methods:**

Considering a group sequential design with one interim analysis for early stopping for efficacy, we conduct a simulation study to evaluate existing analysis techniques when the treatment effect on a continuous outcome is partially or fully mediated by a continuous intermediate variable measuring a casual mechanism. We study the probability of rejecting the null hypotheses on the total effect (i.e., intention-to-treat effect), direct effect and indirect effect, respectively. We examine the bias of maximum likelihood estimator for these effects. We investigate if the penalized (and conditional) maximum likelihood estimator has smaller bias than the maximum likelihood estimator when a trial stopped (did not stop) early.

**Results:**

The presence of an intermediate variable reduces the power of a group sequential design when sample size calculation ignores the causal mechanism, though type I error control remains unaffected. The maximum likelihood estimator is unbiased only for the mediator-outcome path, impacting the properties of mediation analysis since existing methods typically rely on it to estimate the pathways. The penalized maximum likelihood estimator for other pathways has similar bias to the stage-one maximum likelihood estimator, while the conditional maximum likelihood estimator shows negligible or smaller bias than the usual maximum likelihood estimator for estimating the total and the direct effects only.

**Conclusions:**

Mediation analysis needs additional consideration in group sequential designs. As with fixed trial designs, the sample size calculation of group sequential designs should account for the total variability underlying a causal mechanism when the treatment effect is hypothesized to be mediated by an intermediate variable, or risk the overall power to detect an intention-to-treat (total) effect being lower than the nominal value. We suggest reporting several estimators and acknowledging that they may be biased for some mediation pathways. More research is needed to develop methods for the analysis of indirect effect under group sequential designs.

**Supplementary Information:**

The online version contains supplementary material available at 10.1186/s12874-025-02714-y.

## Introduction

Group sequential designs allow a study to stop early as evidence from the trial emerges, without risking the control of the type I error. It is known that the maximum likelihood estimator produces biased estimates of treatment effects if the group sequential design is ignored. Alternative estimators have been proposed [1, 2] but none of them are universally best with respect to their properties when evaluated over a range of treatment effects [3].

To our knowledge, the literature on this design (and more broadly adaptive designs) is focused on settings where the efficacy of an intervention is manifested directly on one or more endpoints. This assumption may not hold in some clinical areas where there is a mechanism of change in which the intervention affects the outcome partly or solely via an intermediate variable.

For instance, the FeelingSafe study was a two-arm study that compared a theoretically driven cognitive therapy with befriending for the treatment of persistent persecutory delusions [5]. The primary endpoint was change in self-reported persecutory delusions at 6 month from baseline. Secondary analyses include testing whether changes in the hypothesised delusion maintenance factors, such as worry, insomnia and safety-seeking behaviours, mediated the change in delusions.

The intermediate variable considered here is known as a “mediator”. Mediation analysis can evaluate a causal mechanism that involves mediators, which concerns with the pathways by which effects arise, and is distinct from the prediction of treatment effects on surrogate endpoints [6]. The literature on mediation analysis focuses on estimating and testing the indirect effect of an intervention (or exposure in non-randomized studies), which quantifies how much of the treatment effect acts through the mediator. A methodological systematic review on the conduct and reporting of mediation analysis found that most randomized controlled trials (published between 1/1/2017 and 1/12/2018) did not report a sample size calculation for mediation analysis [7]. Since then a guideline for reporting mediation analyses of randomized trials [8] and some online calculators for sample size and power calculation for mediation [9, 10] have become available.

To our knowledge, nobody has explored the properties of mediation analysis when the data generation process is stochastic, as in adaptive designs where there is uncertainty in the sample size of the final data. The RAPID trial [11] (which is on-going at the time of writing) is an example of a multi-arm multi-stage trial that has a plan to evaluate putative mediators, as described in the online study protocol.

To fill the gap, we explore the properties of inference when outcome follows a causal mechanism under the setting of a group sequential design with one interim analysis to allow for early stopping for efficacy. We conduct a simulation study to evaluate the operating characteristics of this design and the conditional bias of some commonly proposed estimators. We explore the relation between a causal mechanism and the power/sample size calculation. Where appropriate, we make comparison with the inference of fixed designs that do not incorporate any interim analyses.

## Overview

We review the setting of group sequential designs, the analysis models that describe a causal mechanism, some hypothesis testing methods in mediation analysis and some commonly proposed estimators.

### Group sequential design

A group sequential design consists of the design aspect of a fixed design and stopping boundaries. Let $Z_k$ denote the standardized test statistics at stage $k=1,..., K$, which has the distribution $Z_k \sim N(\theta \sqrt{I_k}, 1)$ where $\theta$ is the treatment effect and $I_k$ is the information level at stage *k*. The computation of the stopping boundaries relies on the distribution of $(Z_1,... , Z_K)$, which is a multivariate normal distribution with $cov (Z_k, Z_l)=\sqrt{I_k/I_l}$ for $1 \le k \le l \le K$. Specifically, for a two-sided test where the null hypothesis is $H_0: \theta =0$ and the alternative hypothesis is $H_1: \theta \ne 0$, the efficacy stopping boundaries, $b_k$, are computed such that the overall type I error rate is controlled at $\alpha$ level when the null hypothesis is rejected at stage *k* with $|Z_k|> b_k$. For non-normal outcomes, asymptotic approximation is often employed such that the general framework of the group sequential design can be implemented [13].

### Causal mechanism of intervention on outcome

Let *M* be a continuous mediator, *X* be a binary variable with $X=1$ if a patient is randomized to the experimental arm and $X=0$ otherwise, and *Y* be the primary continuous outcome. Following the notation in the literature on mediation analysis, a causal mechanism by which an intervention may change the outcome via a mediator can be described by:

1 $$\begin{aligned} M & = i_m + a X + \epsilon _m \end{aligned}$$

2 $$\begin{aligned} Y & = i_y + c' X + b M + \epsilon _y \end{aligned}$$

assuming the absence of interaction terms. The parameters $i_m$ and $i_y$ are the model intercepts, parameter *a* describes the relation between *X* and *M*, parameter *b* describes the relation between *M* and *Y* adjusting for *X*, and parameter $c'$ describes the relation between *X* and *Y* adjusting for *M*. The error terms $\epsilon _m$ and $\epsilon _y$ reflect the variability in *M* that is not explained by *X* and the variability in *Y* that is not explained by its relations with *X* and *M*, respectively.

Assume $\epsilon _m$ and $\epsilon _y$ are independent. An indirect effect (i.e., the effect of *X* on *Y* that is mediated by *M*) is defined as the product of coefficients *a* and *b*. A direct effect of *X* on *Y* that is not mediated through *M* is represented by $c'$. The total effect is equivalent to the intention-to-treat effect considered in trials, which is denoted by $\theta$ above and corresponds to the estimated effect when regressing *Y* on *X* in a simple linear model without *M*. For continuous *M* and *Y*, the total effect can be defined as the sum of the direct and indirect effect [14], i.e., $\theta =a b + c'$.

### Testing in mediation analysis

Null hypotheses in simple mediation analysis are:

- $$H_{01}: \theta =0$$
- $$H_{02}: c'=0$$
- $$H_{03}: ab=0$$

corresponding to the null effect of total, direct and indirect effect, respectively. Note that $H_{01}$ is the primary hypothesis considered in the sample size calculation of trial designs. The other two hypotheses are commonly considered in the secondary analysis without adjustment for multiplicity, i.e., test $H_{02}$ and $H_{03}$ at $\alpha$ level each.

For studies with fixed designs, the maximum likelihood estimator for $\theta$ and $c'$ are unbiased and normally distributed. A T-test can be applied to test $H_{01}$ and $H_{02}$, respectively. Since the distribution of $\hat{a}\hat{b}$, i.e., the estimator of *ab*, is non-normal, several approaches have been proposed to test $H_{03}$, such as the Sobel test [15, 16], joint significance test [17] and Monte Carlo confidence interval test [18, 19].

### Estimators for group sequential designs

For group sequential designs, it is known that the maximum likelihood estimator for the total effect $\theta$ is conditionally and unconditionally biased [13, 20]. This means the expected value of the estimates can over- or under- estimate the true $\theta$ when evaluated for repeated experiments that stop at stage *k* (i.e., conditional bias) and when evaluated for all repeated experiments that stop at any possible stages (i.e., unconditional bias) [21, 22], respectively. For two-stage trial designs with early stopping rules for efficacy, the maximum likelihood estimator has an upward (downward) bias when the study stopped at stage one (continued to the maximum planned sample size) [23–25]. The opposite trend was observed for designs with futility stopping [26]. Readers who are interested in the analytical expressions of conditional and unconditional bias are referred to the cited papers or [3].

Many alternative estimators have been proposed: mean-unbiased estimator, median-unbiased estimator, bias-reduced estimator and estimator based on resampling approach, see for example [1, 2] and the references therein. Yet there is no estimator that is universally unbiased, e.g., the uniform minimum variance unbiased estimator is unconditionally unbiased but it often has large conditional bias [3]. Estimators for secondary endpoints have also been explored [21, 27–29] but not in the context of mediation analysis.

## Method

To our knowledge, nobody has evaluated the quality of inference when the outcome data is generated from the mediation analysis models under group sequential designs. Considering a group sequential design with one interim analysis to allow for early stopping for efficacy, we conduct simulation studies to examine the performance of some testing procedures and the conditional bias of some estimators. Hereafter we refer to this design as $D_g$ and a fixed design without interim analysis as $D_f$.

### Simulation setup: group sequential design and mediation

**Design setup** We set *n* subjects to have $X_i=0$ and *n* subjects to have $X_i=1$ without incorporating a randomization step in the simulation, where *n* is the maximum sample size of an arm in $D_g$. This is analogous to implementing a block randomization procedure of size two. We consider conducting an interim analysis after 50% of the total sample size have the primary outcome, i.e., after *n*/2 subjects in both arms have their outcomes. We use the *gsbounds* command in Stata 18 to compute the Pocock efficacy boundaries, which gives $b_1=b_2=2.1783$ for $\alpha =5\%$. We describe the considered *n* and the number of trial replications when discussing the results for ease of exposition.

**Target of inference** We compute the statistics to test $H_{01}$, $H_{02}$ and $H_{03}$, respectively, and compute the estimates of *a*, *b*, $c'$ and $\theta$ whenever a trial stops.

**Interim analysis** We fit

$$\begin{aligned} E( Y_i ) = \hat{\beta }_0 + \hat{ \theta } X_i \end{aligned}$$

to the data of *n*/2 subjects who are in the control and the intervention arms respectively. The trial is stopped for efficacy when the t-statistic, $| \frac{\hat{\theta }}{\sqrt{ var(\hat{\theta })}}| \ge b_1$, otherwise the trial continues to stage two.

**Final analysis** In addition to fitting the simple model, we fit models (1) and (2) and test $H_{01}$, $H_{02}$ and $H_{03}$ using

- only the stage one data when the trial stops early for efficacy
- all data otherwise.

We test $H_{01}$ using the group-sequential boundaries. For secondary analysis, $H_{02}$ and $H_{03}$ are tested at 5% significance level each regardless of stopping stage. We apply the first and second order delta methods [15, 16], joint significance test [17] and Monte Carlo confidence interval test [18, 19], respectively. See the Supplementary Document for more details.

For the estimation of $\theta , a, b$, and $c'$, we use the stage one data to compute the

- usual maximum likelihood estimator
- penalized maximum likelihood estimator[25]

when the trial stops early for efficacy. We denote these by $\hat{\beta }_{mle.1}$ and $\hat{\beta }_{pmle}$, respectively.

Otherwise, we use

- all data to compute the usual maximum likelihood estimator
- stage two data only to compute the usual maximum likelihood estimator
- all data to compute the maximum likelihood estimator conditional on stopping at stage two (See [21–23] for the mathematical details.)

We denote these by $\hat{\beta }_{mle.all}$, $\hat{\beta }_{mle.2}$ and $\hat{\beta }_{cmle}$, respectively.

We obtain maximum likelihood estimates from the corresponding regression models. We compute $\hat{\beta }_{pmle}$ using the R code provided by [25] and $\hat{\beta }_{cmle}$ by [23].

**Performance measures** For $D_g$, we examine the rejection rate of each hypothesis at stage one and two respectively. The rate corresponds to the power when the corresponding parameter has a non-zero value, otherwise it corresponds to the type I error rate. The sum of the rejection rate at stage one and two corresponds to the overall power/type I error rate.

We examine the stage one conditional bias for $\hat{\beta }_{mle.1}$ and $\hat{\beta }_{pmle}$, respectively, and the stage two conditional bias for $\hat{\beta }_{mle.all}$, $\hat{\beta }_{mle.2}$ and $\hat{\beta }_{cmle}$, respectively. Readers who are interested in the comparison of the root mean squared error of these estimators are referred to the relevant work [3, 23, 25], who considered $D_g$ under some simple data generating mechanisms.

**Data generating mechanisms** We simulate data of size 2*n* according to models (1) and (2) with *a*, *b*, and $c'$ taking value of 0, 0.14, 0.39 or 0.59, $\epsilon _m\sim N(0, \sigma ^2_m)$ and $\epsilon _y\sim N(0, \sigma ^2_y)$, with $\sigma ^2_y=\sigma ^2_m=1$ for all investigation, and $\sigma _m=1.5$ for specific scenarios. The considered magnitudes correspond to zero, small, medium, and large effect sizes in mediation [30]. Without lost of generality, we set $i_m=i_y=-0.4$. Some R codes are available in the Supplementary Document.

### Results

We focus on the characteristics of hypothesis tests and the bias of estimators. Numerical values of the presented plots are available in the Supplementary Document.

#### Hypothesis test about total effect

Consider $D_g$ with $n=191$. The top panel of Table 1 shows the type I error rate for scenarios where $\theta =0$ with $c'=0$, some zero/non-zero *a* and *b* and $\sigma _m=1,1.5$, respectively. The numerical type one error rate ranges from 0.0477 to 0.0569 when either *a* or *b* or both are zeroes. These values are close to the 95% prediction interval of the simulated type I error rate with 10,000 simulation runs. This shows the error rate control for testing $H_{01}:\theta =0$ is not affected by the presence of a mediator in the data generation; the design framework for testing the intention-to-treat effect, $\theta$, can control the type I error rate at the nominal level.

**Table 1:** Probability of rejecting $H_{01}:\theta =0$ when $c^{\prime }=0$

	$\varvec{a=0}$	$\varvec{a=0.14}$	$\varvec{a=0.39}$	$\varvec{a=0.59}$	$\varvec{a=0}$	$\varvec{a=0}$	$\varvec{a=0}$
	$\varvec{b=0}$	$\varvec{b=0}$	$\varvec{b=0}$	$\varvec{b=0}$	$\varvec{b=0.14}$	$\varvec{b=0.39}$	$\varvec{b=0.59}$
$\sigma _m=1$	0.0512	0.0563	0.052	0.0524	0.0511	0.0569	0.0477
$\sigma _m=1.5$	0.0512	0.0493	0.0505	0.0524	0.0518	0.0493	0.0493

The 95% prediction interval of the simulated type I error rate of 5% over 10,000 simulation runs is (0.0457, 0.0543). $D_g$ has the maximum sample size per arm of $n=191$

This $D_g$ with $n=191$ has 90% power to detect $\theta = 0.3481$ at 5% significance level. The top panel of Table 2 shows the overall probability of rejecting $H_{01}$ when $\theta =a b + c' = 0.3481$, computed from 10,000 trial replications. We find the numerical power is close to 90% when $b=0$ and $c'=0.59^2=0.3481$, regardless of the values of *a* and $\sigma _m$. When $a=0, b \ne 0$ and $\theta =c'=0.59^2$, the power ranges from 1% −22% smaller than expected; the loss in power increases with the magnitude of $\sigma _m$ and *b*. These indicate that the sample size calculation may not have captured the variability in the data accurately when $b \ne 0$.

**Table 2:** Overall probability of rejecting $H_{01}:\theta =0$

	$\varvec{a=0}$	$\varvec{a=0.14}$	$\varvec{a=0.39}$	$\varvec{a=0.59}$	$\varvec{a=0}$	$\varvec{a=0}$	$\varvec{a=0}$	$\varvec{a=0.59}$
	$\varvec{b=0}$	$\varvec{b=0}$	$\varvec{b=0}$	$\varvec{b=0}$	$\varvec{b=0.14}$	$\varvec{b=0.39}$	$\varvec{b=0.59}$	$\varvec{b=0.59}$
	$\varvec{c}^{\varvec{\prime }} \varvec{=0.59}^{\varvec{2}}$	$\varvec{c}^{\varvec{\prime }}\varvec{=0.59}^{\varvec{2}}$	$\varvec{c}^{\varvec{\prime }}\varvec{=0.59}^{\varvec{2}}$	$\varvec{c}^{\varvec{\prime }}\varvec{=0.59}^{\varvec{2}}$	$\varvec{c}^{\varvec{\prime }}\varvec{=0.59}^{\varvec{2}}$	$\varvec{c}^{\varvec{\prime }}\varvec{=0.59}^{\varvec{2}}$	$\varvec{c}^{\varvec{\prime }}\varvec{=0.59}^{\varvec{2}}$	$\varvec{c}^{\varvec{\prime }}\varvec{=0}$
$\sigma _m=1$	0.9046	0.9016	0.9072	0.9022	0.8919	0.8474	0.7883	0.7999
$\sigma _m=1.5$	0.9046	0.9014	0.9026	0.9022	0.8884	0.7988	0.6719	0.6736

$D_g$ has the maximum sample size per arm of $n=191$

Considering an adjusted effect size,

3 $$\begin{aligned} \frac{ ab+c'}{ \sqrt{(b^2 \sigma ^2_m + \sigma ^2_y)} } \end{aligned}$$

which was proposed for fixed trial designs with a causal mechanism[12], we further compute the nominal power given $n=191$ using the function *getPowerMeans* in the R package *rpact* [31] for the considered scenarios with $b \ne 0$. Table 3 shows the adjusted effect sizes, nominal powers and 95% prediction interval of the nominal power over 10,000 simulation runs for scenarios with $\sigma _m=1$ and $\sigma _m=1.5$, respectively. Comparing the corresponding values to Table 2, we see that the simulated powers are within (or close to) the 95% prediction interval of the nominal power, suggesting that one shall consider the adjusted effect size (3) for the sample size calculation of group sequential designs when $b \ne 0$. This confirms the required sample size of a group sequential design can be computed on the adjusted effect size to achieve the required power without further adjustment.

**Table 3:** Adjusted total effect size and the corresponding nominal power with 95% prediction interval of the nominal power over 10,000 simulation runs

	$\varvec{\sigma }_{\varvec{m}}\varvec{=1}$				$\varvec{\sigma }_{\varvec{m}}\varvec{=1.5}$			
	Adjusted	Nominal	Lower	Upper	Adjusted	Nominal	Lower	Upper
	total effect	power	limit	limit	total effect	power	limit	limit
$a=0, b=0.14, c^{\prime }=0.59$	0.3447	0.8930	0.8870	0.8991	0.3407	0.8856	0.8794	0.8919
$a=0, b=0.39, c^{\prime }=0.59$	0.3243	0.8524	0.8455	0.8594	0.3005	0.7935	0.7856	0.8015
$a=0, b=0.59, c^{\prime }=0.59$	0.2998	0.7917	0.7838	0.7997	0.2607	0.6698	0.6606	0.6790
$a=0.59, b=0.59, c^{\prime }=0$	0.2998	0.7917	0.7838	0.7997	0.2607	0.6698	0.6606	0.6790

#### Secondary analysis: hypothesis test about the direct effect

We examine the result of testing $H_{02}: c'=0$ from the same design and scenarios as those in Section 3.2.1. Table 4 shows that the numerical type I error rates are over the 95% prediction interval of the type I error rate, with one exceptional scenario where $a=0$, $b=0.59$ and $\sigma _m=1.5$. Table 5 shows the power of testing $H_{02}: c'=0$. We find the power decreases (minimally) with the magnitude of *a* or *b* when one of them is zero.

**Table 4:** Probability of rejecting $H_{02}:c^{\prime }=0$ when $c^{\prime }=0$

	$\varvec{a=0}$	$\varvec{a=0.14}$	$\varvec{a=0.39}$	$\varvec{a=0.59}$	$\varvec{a=0}$	$\varvec{a=0}$	$\varvec{a=0}$
	$\varvec{b=0}$	$\varvec{b=0}$	$\varvec{b=0}$	$\varvec{b=0}$	$\varvec{b=0.14}$	$\varvec{b=0.39}$	$\varvec{b=0.59}$
$\sigma _m=1$	0.0672	0.0745	0.0654	0.0671	0.0691	0.0691	0.0584
$\sigma _m=1.5$	0.0672	0.065	0.0682	0.0688	0.0697	0.0603	0.0529

The 95% prediction interval of the simulated type I error rate of 5% over 10,000 simulation runs is (0.0457, 0.0543). $D_g$ has the maximum sample size per arm of $n=191$

**Table 5:** Overall probability of rejecting $H_{02}:c^{\prime }=0$ at 5% significance level

	$\varvec{a=0}$	$\varvec{a=0.14}$	$\varvec{a=0.39}$	$\varvec{a=0.59}$	$\varvec{a=0}$	$\varvec{a=0}$	$\varvec{a=0}$	$\varvec{a=0.59}$
	$\varvec{b=0}$	$\varvec{b=0}$	$\varvec{b=0}$	$\varvec{b=0}$	$\varvec{b=0.14}$	$\varvec{b=0.39}$	$\varvec{b=0.59}$	$\varvec{b=0.59}$
	$\varvec{c}^{\varvec{\prime }} \varvec{=0.59}^{\varvec{2}}$	$\varvec{c}^{\varvec{\prime }}\varvec{=0.59}^{\varvec{2}}$	$\varvec{c}^{\varvec{\prime }}\varvec{=0.59}^{\varvec{2}}$	$\varvec{c}^{\varvec{\prime }}\varvec{=0.59}^{\varvec{2}}$	$\varvec{c}^{\varvec{\prime }}\varvec{=0.59}^{\varvec{2}}$	$\varvec{c}^{\varvec{\prime }}\varvec{=0.59}^{\varvec{2}}$	$\varvec{c}^{\varvec{\prime }}\varvec{=0.59}^{\varvec{2}}$	$\varvec{c}^{\varvec{\prime }}\varvec{=0}$
$\sigma _m=1$	0.9333	0.9272	0.9122	0.8871	0.9272	0.9147	0.9106	0.0516
$\sigma _m=1.5$	0.9333	0.9305	0.9237	0.9133	0.9248	0.9101	0.9066	0.0509

$D_g$ has the maximum sample size per arm of $n=191$

#### Secondary analysis: hypothesis test about indirect/mediated effect

We examine the results of testing $H_{03}: ab=0$. For the setup considered in Tables 2 and 5, the power of testing $H_{03}$ when $a=b=0.59$ and $c=0$ is close to one. This finding is consistent with the finding that under $D_f$, the power of testing $H_{03}$ is higher than that of $H_{01}$ when the total and indirect effects have the same value [32]. For scenarios where either *a* or *b* or both are zeroes, we obtain type one error rates that range from 0 to 0.052 (see Table 6), which are similar to those in the literature of $D_f$[17, 33].

**Table 6:** Probability of rejecting of rejecting $H_{03}: ab =0$ when $D_g$ has the maximum sample size of n=191 per arm

	$\varvec{a=0}$	$\varvec{a=0.14}$	$\varvec{a=0.39}$	$\varvec{a=0.59}$	$\varvec{a=0}$	$\varvec{a=0}$	$\varvec{a=0}$
	$\varvec{b=0}$	$\varvec{b=0}$	$\varvec{b=0}$	$\varvec{b=0}$	$\varvec{b=0.14}$	$\varvec{b=0.39}$	$\varvec{b=0.59}$
	When $\sigma _m=1$						
Monte Carlo bootstrap CI	0.0012	0.0095	0.0472	0.0498	0.0304	0.0519	0.0535
First order Sobel test	0.0002	0.0015	0.0201	0.0366	0.0088	0.0443	0.0504
Second order Sobel test	0.0002	0.0010	0.0170	0.0342	0.0068	0.0432	0.0499
Joint significance test	0.0018	0.0134	0.0495	0.0487	0.0363	0.0514	0.0525
	When $\sigma _m=1.5$						
Monte Carlo bootstrap CI	0.0012	0.0054	0.0270	0.0469	0.0472	0.0518	0.0545
First order Sobel test	0.0002	0.0005	0.0063	0.0220	0.0241	0.0484	0.0522
Second order Sobel test	0.0002	0.0003	0.0048	0.0184	0.0209	0.0476	0.0517
Joint significance test	0.0018	0.0082	0.0325	0.0476	0.0483	0.0515	0.0532

**Fig. 1:**
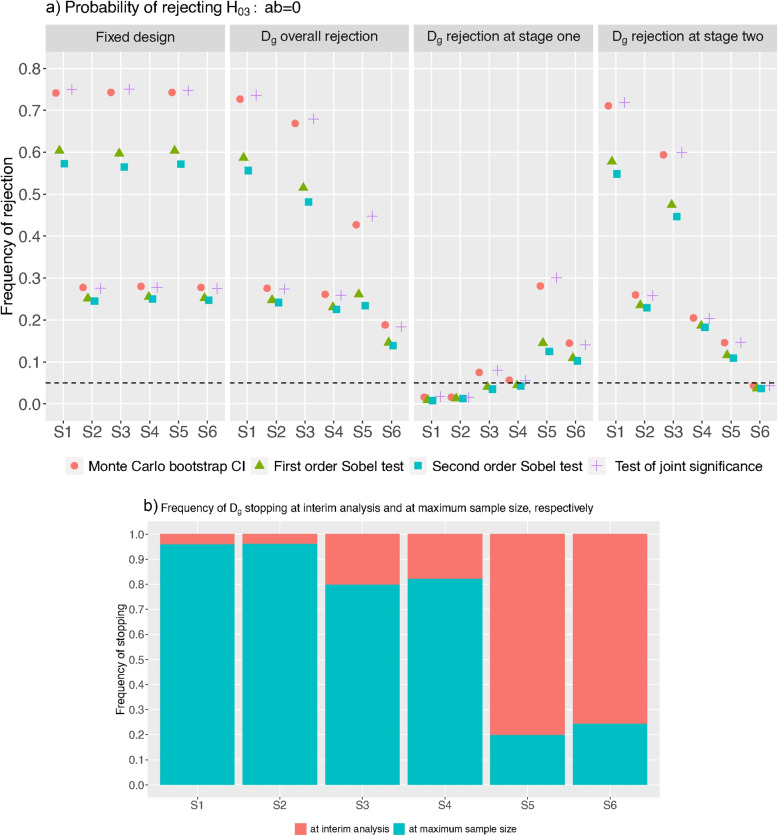
Fixed trial design has 191 subjects per arm. $D_g$ has the maximum sample size of $n=191$. **a** Probability of rejecting $H_{03}$. **b** Frequency of $D_g$ stopping at interim analysis and at the maximum sample size. $D_g$ ($D_f$) has the maximum (fixed) sample size of $n=191$. The values of *a*, *b* and $c'$ for each scenario on the *x*-axis are presented in Supplementary Document Table S1

We consider other scenarios, labelled S1-S6 in what follows, with selected values of *a*, *b* and $c'$ (see Supplementary Document Table S1) using the same design to examine the results of testing $H_{03}$ when $\sigma _m=1$. Figure 1a shows the power of $D_f$, the overall rejection rate, rejection rate at stage one and two of $D_g$, respectively. Figure 1b shows the frequency of stopping the trial at different time points.

Overall, we find the Sobel tests (both first and second order) are less powerful than the test of joint significance and the Monte Carlo bootstrap confidence interval test; the latter two procedures have similar power of rejecting $H_{03}$. This finding is consistent with the observation for studies with fixed sample size [33, 34].

Moreover, for $D_f$, we see that the power of the tests are consistent across scenarios with the same magnitude of *a*, *b* but different $c'$, e.g., comparing those in scenario S1, S3 and S5. This is not the case for $D_g$; the power decreases with the magnitude of $c'$. For example, the power of the joint significance test to detect $a b=0.39 \times 0.14$ reduces from 73.61% to 44.77% when $c'$ increases from 0 to 0.39, i.e., comparing scenario S1 and S5. This might be explained by the fact that when $c'$ is larger in scenario S5, $D_g$ stopped more frequently at stage one than at stage two (8016/10000=0.8016 vs 1984/10000=0.1984), leading to less data for rejecting $H_{03}$ at stage one and less rejection at stage two over the replications, when compared to the high frequency of continuing to stage two under scenario S1 (which has more data and more frequency to test $H_{03}$ at stage two). This explanation is supported by the rejection rate at stage one and stage two shown in Fig. 1a and the stopping frequency in Fig. 1b.

#### Estimation of effects

We examine the properties of some estimators for *a*, *b*, *c* and $\theta$, respectively, with $D_g$ that has $n=347$. We consider scenarios S1-S6 (see Supplementary Document Table S1) with $\sigma _m=1$ and 50,000 trial replications in the simulation. This sample size can detect a standardized effect size of 0.26 with 90% power and 5% type one error rate, using the Pocock stopping boundary for efficacy. We consider a larger number of replications so that there are at least 1000 replications that the trial stops at either stage one or at stage two for evaluation (reported in Supplementary Document Table S2). We consider a larger sample size to minimize the number of replications that there are computational issues with $\hat{\beta }_{pmle}$, which were discarded when computing the property of the estimators.

**Fig. 2:**
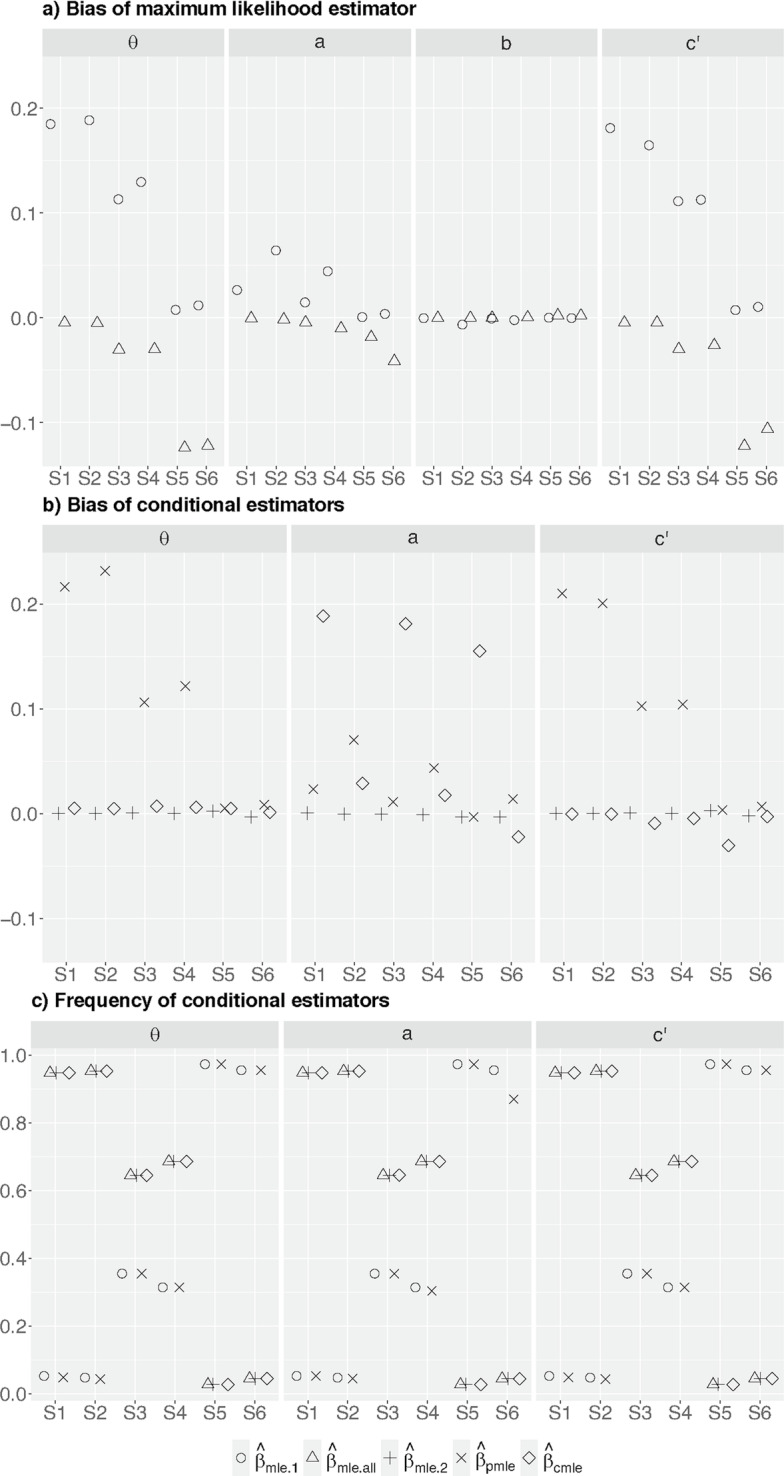
$D_g$ with $n=347$. **a** Bias of the maximum likelihood estimators. **b** Bias of the conditional estimators. **c** The frequency which the estimator was averaged across

Figure 2a shows the conditional bias of $\hat{\beta }_{mle.1}$ and $\hat{\beta }_{mle.all}$. As expected, $\hat{\beta }_{mle.1}$ is overestimating while $\hat{\beta }_{mle.all}$ is underestimating the true values of $\theta$, with $\hat{\beta }_{mle.1}$ having a larger absolute bias than $\hat{\beta }_{mle.all}$. The same pattern of observation applies to the estimation of *a* and $c'$, respectively. The absolute bias of these estimators for $c'$ is similar or slightly smaller than that of $\theta$, while the bias of these estimators for *a* is much lower.

We find $\hat{\beta }_{mle.1}$ and $\hat{\beta }_{mle.all}$ for *b* is unbiased. The early stopping rule does not affect these estimates as the relation between the mediator and outcome is unmodified by the intervention.

**Fig. 3:**
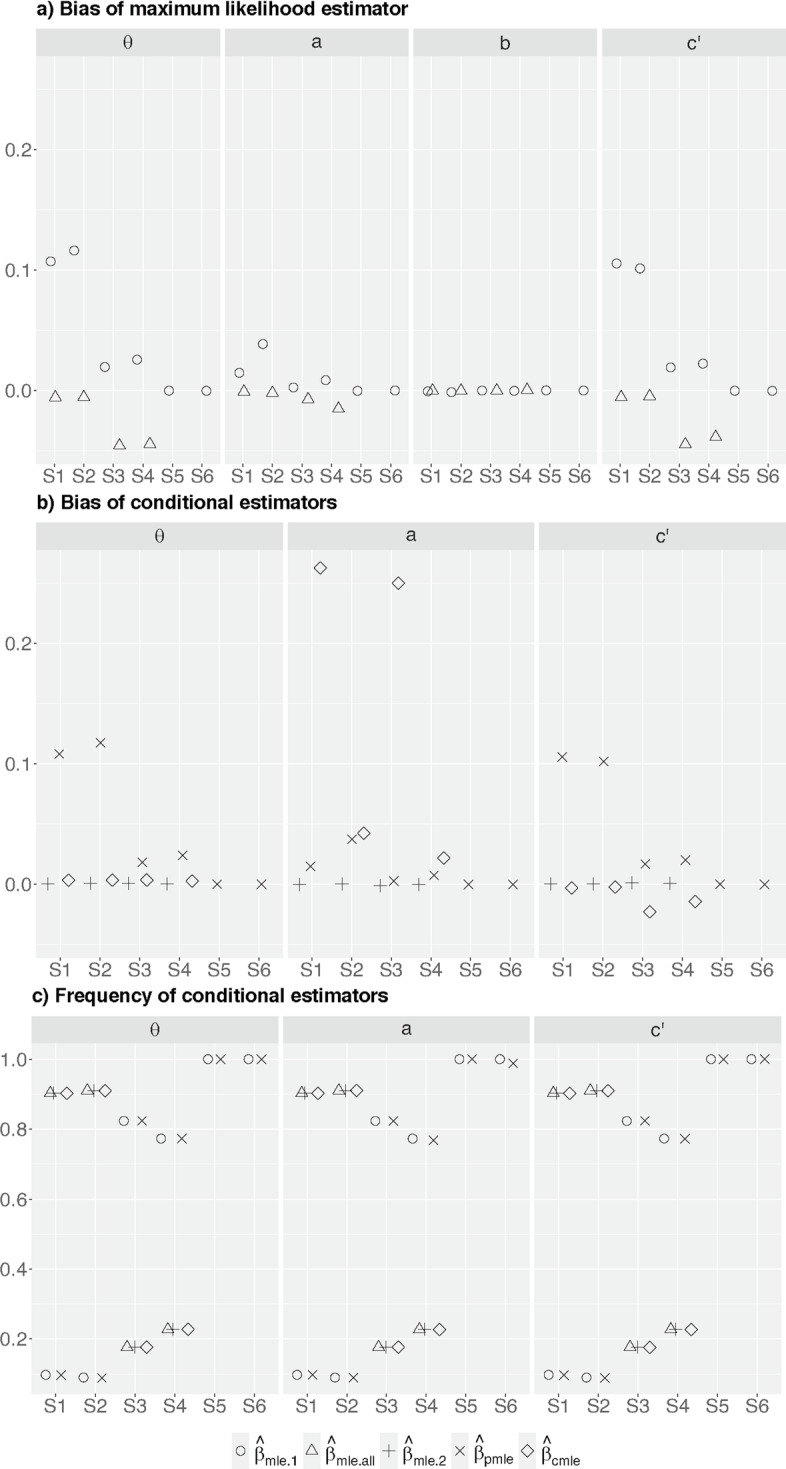
$D_g$ with $n=347*3=1041$. **a** Bias of the maximum likelihood estimators. **b** Bias of the conditional estimators. **c** The frequency which the estimator was averaged across. S5 and S6 only have $\hat{\beta }_{mle.1}$ and $\hat{\beta }_{pmle}$ as the trial always stopped at stage one for efficacy

Figure 2b shows the conditional bias of $\hat{\beta }_{mle.2}$, $\hat{\beta }_{pmle}$, and $\hat{\beta }_{cmle}$, respectively. As expected, $\hat{\beta }_{mle.2}$ computed using stage two data only is unbiased for all parameters. Conditional on stopping at stage one, we find the bias of $\hat{\beta }_{pmle}$ for $\theta , a, c'$ are comparable to that of $\hat{\beta }_{mle.1}$ for most scenarios except under scenarios S1 and S2, where the bias of $\hat{\beta }_{pmle}$ is larger than that of $\hat{\beta }_{mle.1}$. Figure 2c shows these two scenarios have about 5% chance of stopping at stage one. The increased bias of $\hat{\beta }_{pmle}$ might be due to small sample size [25], as the magnitude reduces when the total sample size is three times larger (i.e. compare the crosses in Fig. 2b with those in Fig. 3b; numerical values are available in the Supplementary Document).

Conditional on stopping at stage two, $\hat{\beta }_{cmle}$ for $\theta$ has a negligible bias for all scenarios while $\hat{\beta }_{cmle}$ for $c'$ has a negligible or smaller bias than $\hat{\beta }_{mle.all}$ if there is any. $\hat{\beta }_{cmle}$ for *a* has a positive bias; the absolute magnitude is larger than the corresponding $\hat{\beta }_{mle.all}$ under all the considered scenarios except S6, where $\hat{\beta }_{cmle}$ has a smaller negative bias than $\hat{\beta }_{mle.all}$. There was about 5% chance of stopping at stage two for scenario S6. The pattern of observations is similar when we have three times larger sample size than the presented scenarios (see Fig. 3).

A summary of all findings is presented on Table 7.

**Table 7:** Summary of simulation findings

	Total effect	Direct effect	Indirect effect
Type I error	Controlled at the nominal rate	Inflated under $D_g$ but not $D_f$	Similar to $D_f$
Power	Smaller than expected	Decreases with *a* (or *b*)	Affected by $c^{\prime }$ under $D_g$ but not $D_f$
$\hat{\beta }_{mle.1}$	Positively biased	Positively biased	Positively biased$^*$
$\hat{\beta }_{mle.all}$	Negatively biased	Negatively biased	Negatively biased$^*$
$\hat{\beta }_{pmle}$	Comparable to $\hat{\beta }_{mle.1}$	Comparable to $\hat{\beta }_{mle.1}$	Comparable to $\hat{\beta }_{mle.1}{}^*$
$\hat{\beta }_{cmle}$	Negligible bias	Negligible/smaller bias than $\hat{\beta }_{mle.all}$	Rarely has smaller bias than $\hat{\beta }_{mle.all}{}^*$

^*^For estimation of *a*. $\hat{\beta }_{mle.1}$ and $\hat{\beta }_{mle.all}$ are unbiased for *b*

## Discussion

Researchers need to understand the properties of mediation analysis under a group sequential design as the use of interim analysis in trials increases, e.g., studies funded by the UK’s National Institute of Health and Social Care Research (NIHR) Efficacy and Mechanism Evaluation programme, may contain both a group sequential design and specifically test for some mechanisms.

Considering the setting when data is generated from mechanistic models (Eqs. (1) and (2)), our simulation findings highlight a caveat in the sample size calculation of group sequential designs. We find that the nominal power is obtained only when the sample size calculation considers an adjusted effect size, as shown in Eq. (3), as in a fixed design when there is an underlying causal mechanism [12]. We note that the presence of a mediator in the data generating mechanism does not affect the sample size/power comparison between a group sequential design and a fixed design. This is because the inflation factor, which is the ratio of the required sample size of a group sequential design to that of a fixed design, is independent of the effect size. This inflation factor is always larger than one, reflecting the proportion of additional sample size that is needed in group sequential designs to uphold the same type I error rate as fixed designs [13]. In our investigation, we have not considered the presence of treatment-mediator interactions. Sensitivity analysis with respect to the required power and sample size may be conducted by simulation study at the design stage when these interactions are of interest.

For the secondary analysis, we considered 5% significance level for the test of the direct effect. We see the inflation of type I error rate in group sequential designs, possibly due to the interim analyses and/or the use of the same outcome data in testing three hypotheses. This is not the case when a fixed design is considered. See Supplementary Document Table S3 for some examples where the type I error rate is controlled at the nominal level (within or close to the 95% prediction interval). The power under the group sequential designs (fixed designs) decreases (minimally) with the magnitude of *a* or *b* when one of them is zero while $c'$ remains the same.

As with fixed designs [17, 33], the type I error of the test of indirect effect is not controlled at the nominal level in our investigation. Nevertheless, the power of the test of indirect effect may vary with the underlying magnitude of direct effect even when the indirect effect has the same magnitude in different studies that used the same group sequential design. As the estimators of *a* are not unbiased, the power of the test of indirect effect may also be spurious. These two points are not pertinent to fixed designs. Future work can explore optimal testing procedures for hypotheses that are related to mediation analysis in adaptive designs, with respect to the control of multiplicity.

We recommend the computation of maximum likelihood estimator for all parameters and supplement with $\hat{\beta }_{pmle}$ or $\hat{\beta }_{cmle}$ (depending on the stopping time) for the estimation of $\theta$ and $c'$. We remind readers that the computation of $\hat{\beta }_{pmle}$ might not converge in some settings, such as studies with small sample sizes. The potential of having biased estimates should be acknowledged when reporting study results. Moreover, when presenting $\hat{\beta }_{cmle}$, one should also note that it has a larger variability than $\hat{\beta }_{mle.all}$ computed at the end of stage two. This is due to the loss of information when conditioning on stopping time [23]. Although not considered here, one can compute the standard deviation of the estimators by bootstrapping but should not compute the confidence interval using these estimates as it will not have the correct coverage for most scenarios [2].

We have only considered a design with a continuous endpoint and early stopping for efficacy. We believe our simulation findings are good indications to other adaptive designs when the data follows the mediation models (Eqs. (1) and (2)):

- The power of adaptive designs is less than expected when the sample size calculation does not account for the presence of a mediator.
- Estimation of *b* is unlikely to be affected by other design adaptations. This is subject to the assumption that there are no unmeasured M-Y confounders (as we have assumed in this work).
- The maximum likelihood estimator for *a*, $c'$ and $\theta$ might have similar estimation properties.

Future work may propose alternative estimators for *a*, explore the computation of confidence interval for the estimates of effects, and explore all these in the setting of other adaptive designs. We note that Eqs. (1) and (2) reflect a simple causal mechanism that we assumed is true. More investigation in the setting of adaptive designs is required for complex mechanisms, such as the presence of multiple mediators in a single mechanism and the presence of mediator-outcome confounding. For example, one may explore how to adapt weighting based approach [35, 36] and g-formula [37] for the latter setting. To our knowledge, nobody has explored the performance of these advanced estimation methods for mediation analysis under adaptive designs. There is also no relevant finding for the settings where the outcome variable and mediator are non-continuous. The finding here is non-transferrable, as $a b + c' \ne \theta$ and the indirect effect depends on the values of the exposure/randomization and mediator in a nonlinear fashion [38]. More investigation for sample size calculations is thus required.

## Conclusion

As with the fixed trial designs, sample size calculation of a group sequential design should account for the underlying causal mechanism when the effect of the intervention is believed to be mediated by a mediator, or risk the overall power to detect an intention-to-treat effect. We suggest to report maximum likelihood estimator for the estimation of all effects and supplement with the penalized (or conditional) maximum likelihood estimator for the total and direct effects when the study stops early for efficacy (did not stop), acknowledging that these estimators are not unbiased. More research is needed for the testing and estimation methods of the indirect effect under the group sequential designs.

## Supplementary Information

Supplementary Material 1

## Data Availability

Code to simulate data and reproduce the results in this paper can be found in the supplementary materials.
