# Supplementary material for: Inference in group sequential designs with causal mechanisms: implications for power and mediation analysis
Source: BMC Med Res Methodol. 2025 Nov 14;25:257. doi: 10.1186/s12874-025-02714-y (PMC12619153; doi:10.1186/s12874-025-02714-y)
Supplement: Supplementary file 1 — Supplementary Material 1 [file 12874_2025_2714_MOESM1_ESM.zip › Supplementary-Documents.pdf]

Supplementary material

Testing methods for indirect effect

Sobel tests

Let $\hat{a}, \hat{b}$ denote the MLE of a and b respectively and $s_{\hat{a}}, s_{\hat{b}}$ the corresponding standard deviation of $\hat{a}$ and $\hat{b}$. Consider normal approximation, the Sobel test uses the test statistic, $\hat{a}\hat{b}/s_{\hat{a}\hat{b}}$, where $s_{\hat{a}\hat{b}} = \sqrt{\hat{a}^2 s_{\hat{b}}^2 + \hat{b}^2 s_{\hat{a}}^2}$ for the first-order approximation and $s_{\hat{a}\hat{b}} = \sqrt{\hat{a}^2 s_{\hat{b}}^2 + \hat{b}^2 s_{\hat{a}}^2 + s_{\hat{a}}^2 s_{\hat{b}}^2}$ for the second-order approximation.

Test of joint significance

The test of joint significance rejects $H_{03} : ab = 0$ at α level only if both the null hypotheses that $a = 0$ and $b = 0$ are rejected each at α level, based on $\hat{a}$, $\hat{b}$, $s_{\hat{a}}$ and $s_{\hat{b}}$ using Student-t distribution with the corresponding degree of freedom, according to the fitted models (1) and (2).

Monte Carlo bootstrap confidence interval test

The Monte Carlo bootstrap confidence interval test approximates the distribution of ab by random sampling from two normal distributions with means $\hat{a}$ and $\hat{b}$ and standard deviations $s_{\hat{a}}$ and $s_{\hat{b}}$, respectively. Specifically, the samples of $\hat{a}$ and $\hat{b}$ are multiplied together to form the distribution of ab, the 2.5th and 97.5th values in the distribution form the 95% confidence interval. H_{03} is rejected at 5% significance level when the 95% confidence interval excludes zero.

Numerical results of simulation study

Table S1: Values in the data generating mechanisms of S1-S6 for evaluating H_{03} and estimation properties.

Scenario	a	b	c'	$ab + c' = \theta$
S1	0.39	0.14	0.00	0.05
S2	0.14	0.39	0.00	0.05
S3	0.39	0.14	0.14	0.19
S4	0.14	0.39	0.14	0.19
S5	0.39	0.14	0.39	0.44
S6	0.14	0.39	0.39	0.44

Table S2: Frequency of estimating an effect by an estimator. The value divided by 50000 corresponds to the point in Figure 2c. Empty cells correspond to scenarios where an estimate is not computed.

Scenario	path	$\hat{\beta}_{mle.1}$	$\hat{\beta}_{pmle}$	$\hat{\beta}_{mle.all}$	$\hat{\beta}_{mle.2}$	$\hat{\beta}_{cmle}$
S1	a	2631	2631	47369	47369	47369
S2	a	2383	2260	47617	47617	47617
S3	a	17740	17740	32260	32260	32260
S4	a	15707	15182	34293	34293	34293
S5	a	48637	48631	1363	1363	1363
S6	a	47757	43482	2243	2243	2243
S1	b	2631		47369		
S2	b	2383		47617		
S3	b	17740		32260		
S4	b	15707		34293		
S5	b	48637		1363		
S6	b	47757		2243		
S1	c'	2631	2441	47369	47369	47369
S2	c'	2383	2168	47617	47617	47617
S3	c'	17740	17738	32260	32260	32260
S4	c'	15707	15704	34293	34293	34293
S5	c'	48637	48637	1363	1363	1363
S6	c'	47757	47757	2243	2243	2243
S1	θ	2631	2441	47369	47369	47369
S2	θ	2383	2168	47617	47617	47617
S3	θ	17740	17738	32260	32260	32260
S4	θ	15707	15704	34293	34293	34293
S5	θ	48637	48637	1363	1363	1363
S6	θ	47757	47757	2243	2243	2243

Table S3: Probability of rejecting $H_{02} : c' = 0$ when D_f has 191 subjects per arm, with $\sigma_m = 1$ in the data generating mechanism and 10,000 replications.

Value of	Type I error		Power when $c' = 0.59^2$	
non-zero a or b	$a = 0, b$ is non-zero	a is non-zero $b = 0$	$a = 0, b$ is non-zero	a is non-zero $b = 0$
0.14	0.0498	0.0483	0.925	0.9186
0.39	0.0504	0.0503	0.9301	0.9118
0.59	0.0508	0.0519	0.9238	0.9043
0.7	0.0513	0.0494	0.92	0.8954
0.8	0.0507	0.0448	0.9231	0.8838
0.9	0.0508	0.0513	0.9269	0.8687

Table S4: Frequency of stopping over 10000 replications for the results in Figure 1.

	S1	S2	S3	S4	S5	S6
Stop at interim analysis	407	393	2025	1783	8016	7572
Continue until the maximum sample size	9593	9607	7975	8217	1984	2428

Table S5: Probability of rejecting $H_{03} : ab = 0$ in Figure 1 by different methods.

Scenario	design	Monte Carlo bootstrap CI	1st order Sobel test	2nd order Sobel test	Test of joint significance
S1	D_f	0.7413	0.6038	0.5727	0.7498
S1	D_g overall	0.7267	0.5870	0.5564	0.7361
S1	D_g at stage 1	0.0158	0.0093	0.0080	0.0172
S1	D_g at stage 2	0.7109	0.5777	0.5484	0.7189
S2	D_f	0.2775	0.2513	0.2449	0.2754
S2	D_g overall	0.2754	0.2476	0.2416	0.2735
S2	D_g at stage 1	0.0157	0.0126	0.0124	0.0156
S2	D_g at stage 2	0.2597	0.2350	0.2292	0.2579
S3	D_f	0.7427	0.5972	0.5646	0.7506
S3	D_g overall	0.6687	0.5149	0.4818	0.6794
S3	D_g at stage 1	0.0749	0.0404	0.0350	0.0799
S3	D_g at stage 2	0.5938	0.4745	0.4468	0.5995
S4	D_f	0.2800	0.2550	0.2503	0.2778
S4	D_g overall	0.2611	0.2303	0.2248	0.2591
S4	D_g at stage 1	0.0563	0.0441	0.0420	0.0553
S4	D_g at stage 2	0.2048	0.1862	0.1828	0.2038
S5	D_f	0.7427	0.6036	0.5718	0.7471
S5	D_g overall	0.4271	0.2609	0.2337	0.4477
S5	D_g at stage 1	0.2811	0.1452	0.1247	0.3007
S5	D_g at stage 2	0.1460	0.1157	0.1090	0.1470
S6	D_f	0.2774	0.2519	0.2468	0.2746
S6	D_g overall	0.1881	0.1463	0.1389	0.1838
S6	D_g at stage 1	0.1447	0.1092	0.1025	0.1407
S6	D_g at stage 2	0.0434	0.0371	0.0364	0.0431

Table S6: Numerical values of bias in Figure 2a and b. Empty cells correspond to scenarios where an estimate is not computed.

Scenario	path	$\hat{\beta}_{mle.1}$	$\hat{\beta}_{pmle}$	$\hat{\beta}_{mle.all}$	$\hat{\beta}_{mle.2}$	$\hat{\beta}_{cmle}$
S1	a	0.0266	0.0234	-0.0003	0.0006	0.1888
S2	a	0.0643	0.0705	-0.0014	-0.0001	0.0292
S3	a	0.0148	0.0113	-0.0043	-0.0003	0.1814
S4	a	0.0444	0.0437	-0.0100	-0.0005	0.0176
S5	a	0.0008	-0.0030	-0.0183	-0.0029	0.1554
S6	a	0.0037	0.0142	-0.0412	-0.0032	-0.0220
S1	b	-0.0003		-0.0001		
S2	b	-0.0064		0.0001		
S3	b	-0.0007		0.0002		
S4	b	-0.0021		0.0005		
S5	b	0.0002		0.0025		
S6	b	-0.0002		0.0022		
S1	c'	0.1811	0.2103	-0.0046	0.0006	-0.0003
S2	c'	0.1647	0.2008	-0.0044	0.0003	-0.0002
S3	c'	0.1114	0.1028	-0.0297	0.0008	-0.0091
S4	c'	0.1127	0.1043	-0.0260	0.0004	-0.0043
S5	c'	0.0075	0.0038	-0.1222	0.0033	-0.0302
S6	c'	0.0105	0.0069	-0.1059	-0.0016	-0.0027
S1	θ	0.1849	0.2166	-0.0047	0.0006	0.0051
S2	θ	0.1886	0.2319	-0.0050	0.0002	0.0049
S3	θ	0.1132	0.1063	-0.0303	0.0007	0.0073
S4	θ	0.1297	0.1218	-0.0299	0.0003	0.0064
S5	θ	0.0077	0.0053	-0.1238	0.0028	0.0051
S6	θ	0.0119	0.0087	-0.1218	-0.0030	0.0015

Table S7: Numerical values of bias in Figure 3a and b. Empty cells correspond to scenarios where an estimate is not computed.

Scenario	path	$\hat{\beta}_{mle.1}$	$\hat{\beta}_{pmle}$	$\hat{\beta}_{mle.all}$	$\hat{\beta}_{mle.2}$	$\hat{\beta}_{cmle}$
S1	a	0.0148	0.0147	-0.0009	-0.0002	0.2623
S2	a	0.0387	0.0373	-0.0019	0.0003	0.0424
S3	a	0.0026	0.0025	-0.0073	-0.0011	0.2497
S4	a	0.0087	0.0072	-0.0151	-0.0004	0.0217
S5	a	-0.0002	-0.0002			
S6	a	0.0001	-0.0004			
S1	b	-0.0006		0.0000		
S2	b	-0.0013		-0.0000		
S3	b	0.0000		0.0001		
S4	b	-0.0002		0.0005		
S5	b	0.0002				
S6	b	0.0000				
S1	c'	0.1054	0.1055	-0.0055	0.0004	-0.0034
S2	c'	0.1014	0.1017	-0.0047	0.0004	-0.0024
S3	c'	0.0192	0.0167	-0.0447	0.0011	-0.0230
S4	c'	0.0224	0.0199	-0.0386	0.0005	-0.0144
S5	c'	-0.0001	-0.0001			
S6	c'	-0.0002	-0.0002			
S1	θ	0.1072	0.1081	-0.0057	0.0003	0.0032
S2	θ	0.1162	0.1174	-0.0055	0.0004	0.0035
S3	θ	0.0196	0.0181	-0.0456	0.0009	0.0035
S4	θ	0.0257	0.0239	-0.0444	0.0003	0.0027
S5	θ	-0.0001	-0.0001			
S6	θ	-0.0002	-0.0002			

Table S8: Frequency of estimating an effect by an estimator. The value divided by 50000 corresponds to the point in Figure 3c. Empty cells correspond to scenarios where an estimate is not computed.

Scenario	path	$\hat{\beta}_{mle.1}$	$\hat{\beta}_{pmle}$	$\hat{\beta}_{mle.all}$	$\hat{\beta}_{mle.2}$	$\hat{\beta}_{cmle}$
S1	a	4877	4877	45123	45123	45123
S2	a	4495	4481	45505	45505	45505
S3	a	41172	41172	8828	8828	8828
S4	a	38642	38411	11358	11358	11358
S5	a	50000	50000			
S6	a	50000	49383			
S1	b	4877		45123		
S2	b	4495		45505		
S3	b	41172		8828		
S4	b	38642		11358		
S5	b	50000				
S6	b	50000				
S1	c'	4877	4825	45123	45123	45123
S2	c'	4495	4440	45505	45505	45505
S3	c'	41172	41172	8828	8828	8828
S4	c'	38642	38642	11358	11358	11358
S5	c'	50000	50000			
S6	c'	50000	50000			
S1	θ	4877	4825	45123	45123	45123
S2	θ	4495	4441	45505	45505	45505
S3	θ	41172	41172	8828	8828	8828
S4	θ	38642	38642	11358	11358	11358
S5	θ	50000	50000			
S6	θ	50000	50000			
